# Anatomical considerations for reducing ocular emergencies during spaceflight

**DOI:** 10.1007/s11845-023-03407-5

**Published:** 2023-05-27

**Authors:** Ethan Waisberg, Joshua Ong, Mouayad Masalkhi, Andrew G. Lee, John Berdahl

**Affiliations:** 1 University College Dublin School of Medicine, Belfield, Dublin, Ireland; 2 Michigan Medicine, University of Michigan, Ann Arbor, USA; 3 Center for Space Medicine, Baylor College of Medicine, Houston, TX USA; 4 Department of Ophthalmology, Blanton Eye Institute, Houston Methodist Hospital, Houston, TX USA; 5 The Houston Methodist Research Institute, Houston Methodist Hospital, Houston, TX USA; 6 Departments of Ophthalmology, Neurology, and Neurosurgery, Weill Cornell Medicine, New York, NY USA; 7 Department of Ophthalmology, University of Texas Medical Branch, Galveston, TX USA; 8 University of Texas MD Anderson Cancer Center, Houston, TX USA; 9 A&M College of Medicine, Bryan, TX USA; 10 Department of Ophthalmology, The University of Iowa Hospitals and Clinics, Iowa City, IA USA; 11 Vance Thompson Vision, Sioux Falls, SD USA

**Keywords:** Acute angle-closure glaucoma, Astronaut, Spaceflight, Vision

## Abstract

**Purpose:**

The privatization of space travel is opening civilian spaceflight to an unprecedented number of individuals now and in the immediate future. The increase in the number and diversity of space travelers will mean increased exposure to both physiologic and pathologic changes observed during acute and prolonged microgravity.

**Aims:**

In this paper, we describe the anatomic, physiologic, and pharmacologic factors to consider that impact acute angle-closure glaucoma risk during spaceflight.

**Conclusions:**

Based on these factors, we elaborate upon areas of medical considerations and provide future recommendations that may aid in reducing the risk of acute angle-closure glaucoma in the next era of spaceflight.

## Introduction

The privatization of space travel is opening civilian spaceflight to an unprecedented number of individuals now and in the immediate future [1]. The increase in the number and diversity of space travelers will mean increased exposure to both physiologic and pathologic changes observed during acute and prolonged microgravity [2]. Rare terrestrial ocular emergency conditions including acute angle-closure glaucoma pose special and unique challenges in space flight. Chronic spaceflight exposure to radiation leading to radiation cataract is another potential risk for precipitating angle closure.

In this paper, we describe the anatomic, physiologic, and pharmacologic factors to consider that impact acute angle-closure glaucoma risk during spaceflight. Based on these factors, we elaborate upon areas of medical considerations and provide future recommendations that may aid in reducing the risk of acute angle-closure glaucoma in the next era of spaceflight.

Several physiological mechanisms have been proposed to explain ocular findings seen in spaceflight. The cephalad redistribution of bodily fluids, particularly the cephalad circulation of cerebral spinal fluid (CSF), that astronauts on the International Space Station (ISS) experience in a microgravity setting is a major factor. Numerous physiological and anatomical alterations are possibly caused by confinement into ocular interstitial spaces. Cerebral edema, a consequence of diverse types of brain tissue injuries, has been used as a paradigm to try to further understand the etiology of the neuro-ocular findings unique to spaceflight. These findings are collectively known as spaceflight associated neuro-ocular syndrome (SANS) and have been hypothesized to occur due to cephalad fluid shift [3, 4]. This constellation of neuro-ophthalmic findings have been noted to be one of the large physiologic barriers to exploration spaceflight such as the mission to Mars [5, 6]. Choroidal expansion occurring in SANS could potentially lead to AACG [7].

Spaceflight or simulated microgravity may also affect a number of other variables related to cerebral autoregulation, including decreases in blood volume and perhaps vascular characteristics [8]. The mechanism most often cited to explain orthostatic sensitivity after spaceflight is the decrease in cerebral blood flow autoregulation brought-on by the lack of the gravitational field [8]. In one rodent study, cerebral arteries demonstrated hypertrophy (media layer) in microgravity [9].

**Fig. 1:**
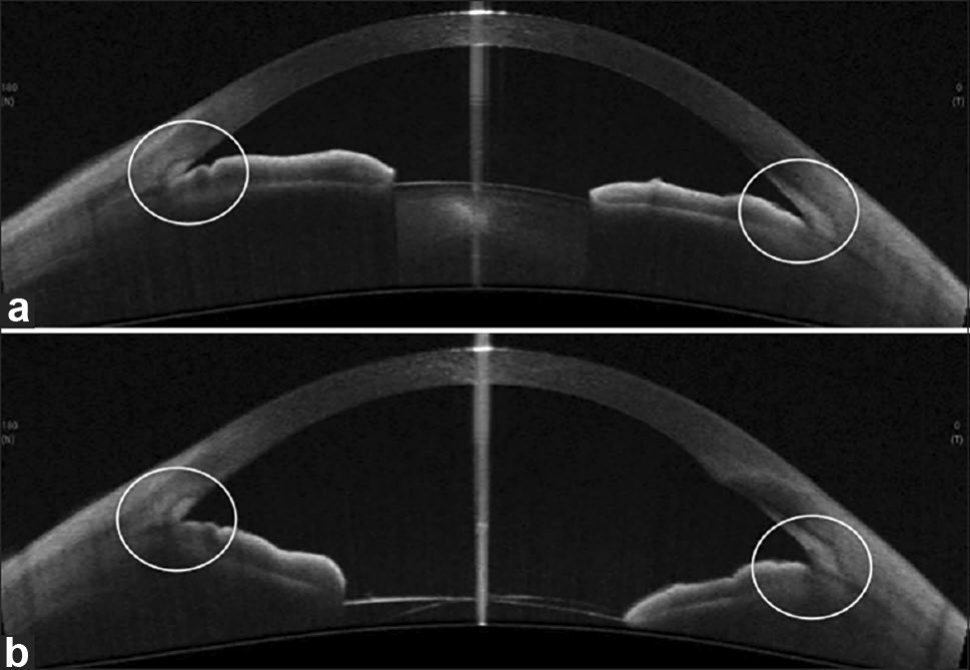
**a** Anterior segment OCT of an angle closure glaucoma with narrow angles (circled). **b** Anterior segment OCT showing wide angles (circled). Both circles contain the trabecular meshwork. Reprinted with permission from Angmo D, Nongpiur ME, Sharma R, Sidhu T, Sihota R, and Dada T. Clinical utility of anterior segment swept-source optical coherence tomography in glaucoma. Oman J Ophthalmol. Under Creative Commons Attribution-NonCommercial-ShareAlike 3.0 License

AACG occurs when aqueous humor outflow is interrupted in the trabecular meshwork, leading to an increase in intraocular pressure (Fig. 1). AACG can be divided into four subtypes: pupillary block, anterior lens subluxation, plateau iris syndrome, and crowded angle which will be described below.

## Pupillary block

The most frequent cause of AACG is called “pupillary block,” which develops when the pupillary portion of the iris and lens becomes functionally blocked between the anterior and posterior chambers [10]. Typically, the iris bends forward (iris bombe) as a result of increased intraocular pressure in the posterior chamber and accompany pupillary block [10].

## Crowded angle

Patients with a crowded angle have thicker iris tissue at the base, leaving less room for the trabecular meshwork to drain. The iris is dragged peripherally and moved into the iridocorneal angle as a result of the contraction of the iris dilator muscles in mydriasis. Pupillary block often happens while the iris is returning to its resting undilated stage after being dilated, which might come from this crowding of tissue and obstruction of the trabecular meshwork [10].

## Anterior lens subluxation

When the anterior lens subluxes, the lens protrudes and either contacts the iris or enters the anterior chamber. Because the zonules anchoring the lens in place are naturally prone to laxity, this might arise. Many conditions (e.g., pseudoexfoliation syndrome, Marfan syndrome, homocystinuria, Ehlers-Danlos syndrome, and Weill-Marchesani syndrome) can lead to zonular laxity, which may all affect ocular structures [10]. Similar to pupillary block, aqueous fluid is unable to drain via the trabecular meshwork because the anterior and posterior chambers are connected but blocked.

## Plateau iris syndrome

An anterior chamber depth that is generally normal, a crowded angle, and a flat iris are all indicative of plateau iris syndrome (PIS). The peripheral iris base of the iris inserts more anteriorly on the ciliary body, resulting in PIS. The iridocorneal angle becomes smaller as a consequence, making it more likely to get blocked during mydriasis. Patients with PIS thus remain at risk for AACG despite having a patent iridotomy.

It is important to note that the greatest anatomical risk factor for angle closure is depth of the anterior chamber [11]. Other risk factors for AACG include an anteriorly positioned lens, short axial length, thick lens, and plateau iris configuration [10]. The risk of drug-induced AACG in space is increased as many of the anticholinesterase or cholinergic medications used by NASA to manage space motion sickness (such as scopolamine and promethazine) cause mydriasis which increases the risk of AACG, due to anterior displacement of lens-iris diaphragm [12]. With space tourism, individuals traveling to space may also be taking a variety of medications which are known as possible AACG precipitants, including α_1_-adrenergic agonists, β_2_ adrenergic agonists, sulfonamides, serotonergic agents, and antihistamines [10]. In addition to these risk factors, a dimly lit environment can also precipitate AACG, which may occur during spaceflight.

Ionizing radiation during long-duration spaceflight may cause cataracts, from exposure to galactic comic radiation or solar particle events. Radiation-induced cataract is a dose-dependent phenomena, and as the ionizing radiation dosage is increased, the lens becomes more opaque [13]. Local changes in the organized arrangement of lens cells caused by oxidation of protein sulfhydryl groups and the creation of high-molecular-weight complexes can lead to phacolytic glaucoma. Thus, cataract is a known risk factor for AACG. Although there have been no reported cases of astronauts developing AACG during or post-spaceflight, one additional rational for protective countermeasures against radiation exposure includes reduced risk for radiation-induced cataracts [14].

While the risks of AACG are rare with an estimated prevalence of 0.5–0.8% and have yet to be clearly defined in spaceflight, if AACG were to occur, the resulting visual loss and ocular pain could impact critical astronaut function and the mission [15]. Secondary optic nerve damage and visual loss if not rapidly treated aggressively and early can be permanent in AACG.

In the case of AACG in space, an immediate reduction in intra-ocular pressure via medical management would be the first step but on Earth depending on etiology a bilateral laser or surgical peripheral iridotomy may be necessary. As laser iridotomy is not available, medication to lower the IOP including topical ophthalmic beta blockers and muscarinic agonists is absolutely critical for future spaceflight.

Lower-body negative pressure (LBNP) is an emerging countermeasure for SANS by mitigating the cephalad fluid shifts in microgravity. However acute usage of LBNP in spaceflight did not impact choroidal thickness, which suggests that choroidal engorgement may occur secondary to cerebral venous congestion [16]. Gonioscopy is the gold standard method to examine the drainage angles and assess AACG risk, which can be performed quickly and with little patient discomfort. For objective measurement of the angle, ultrasound biomicroscopy or Visante OCT (Zeiss, Germany) can also be used for imaging. Further studies are required on the structural and functional effects on ocular structure on long-duration spaceflight [17–19].

Based on these considerations, we recommend that assessment of the angle be included in any ophthalmic screening prior to spaceflight in the era of civilian spaceflight and SANS. This includes a detailed medical history (including risk factors and current medications), optic nerve examination [20], and gonioscopy to identify for anatomical AACG risk factors. As we prepare for a future where spaceflight is more accessible to a civilian population with diverse physiology and anatomy, it becomes increasingly important to take into the considerations to reduce the risk of acute medical risks such as acute, sight-threatening glaucoma.

## References

[1] Stepanek J, Blue RS, Parazynski S (2019) Space medicine in the era of civilian spaceflight. Longo DL, ed. N Engl J Med 380(11):1053–1060. 10.1056/NEJMra1609012 10.1056/NEJMra1609012 30865799

[2] Waisberg E, Ong J, Paladugu P et al (2022) Challenges of artificial intelligence in space medicine. Space: Sci Technol 2022:1–7. 10.34133/2022/9852872

[3] Lee AG, Mader TH, Gibson CR et al (2020) Spaceflight associated neuro-ocular syndrome (SANS) and the neuro-ophthalmologic effects of microgravity: a review and an update. npj Micrograv 6(1):7. 10.1038/s41526-020-0097-9 10.1038/s41526-020-0097-9 PMC7005826 32047839

[4] Ong J, Tavakkoli A, Strangman G et al (2022) Neuro-ophthalmic imaging and visual assessment technology for spaceflight associated neuro-ocular syndrome (SANS). Surv Ophthalmol. Published online April 21, 2022:S0039–6257(22)00048–0. 10.1016/j.survophthal.2022.04.004 10.1016/j.survophthal.2022.04.004 35461882

[5] Patel ZS, Brunstetter TJ, Tarver WJ et al (2020) Red risks for a journey to the red planet: the highest priority human health risks for a mission to Mars. npj Micrograv 6(1):33. 10.1038/s41526-020-00124-6 10.1038/s41526-020-00124-6 PMC7645687 33298950

[6] Waisberg E, Ong J, Lee AG (2023) Factors associated with optic disc edema development during spaceflight. JAMA Ophthalmol. Published online March 16, 2023. 10.1001/jamaophthalmol.2023.0303 10.1001/jamaophthalmol.2023.0303 36928752

[7] Waisberg E, Ong J, Masalkhi M, Lee AG (2023) Optic neuropathy in spaceflight-associated neuro-ocular syndrome (SANS). Ir J Med Sci. Published online April 1, 2023. 10.1007/s11845-023-03353-2 10.1007/s11845-023-03353-2 37004665

[8] Iwasaki K ichi, Levine BD, Zhang R et al (2007) Human cerebral autoregulation before, during and after spaceflight: microgravity and dynamic cerebrovascular control. The J Physiol 579(3):799–810. 10.1113/jphysiol.2006.119636 10.1113/jphysiol.2006.119636 PMC2151354 17185344

[9] Wilkerson MK, Muller-Delp J, Colleran PN, Delp MD (1999). Effects of hindlimb unloading on rat cerebral, splenic, and mesenteric resistance artery morphology. J Appl Physiol.

[10] Yang MC, Lin KY (2019). Drug-induced acute angle-closure glaucoma: a review. Journal of Current Glaucoma Practice.

[11] Yip JLY, Foster PJ (2006). Ethnic differences in primary angle-closure glaucoma. Curr Opin Ophthalmol.

[12] Brooks AMV, West RH, Gillies WE (1986). The risks of precipitating acute angle-closure glaucoma with the clinical use of mydriatic agents. Med J Aust.

[13] Lipman RM, Tripathi BJ, Tripathi RC (1988). Cataracts induced by microwave and ionizing radiation. Surv Ophthalmol.

[14] Shah SS, Meyer JJ (2022) Lens induced glaucoma. In: StatPearls. StatPearls Publishing. Accessed December 23, 2022. http://www.ncbi.nlm.nih.gov/books/NBK574524/34662038

[15] Ramesh S, Maw C, Sutton CJ, Gandhewar JR, Kelly SP (2005). Ethnic aspects of acute primary angle closure in a UK mulicultural conurbation. Eye.

[16] Greenwald SH, Macias BR, Lee SMC (2021). Intraocular pressure and choroidal thickness respond differently to lower body negative pressure during spaceflight. J Appl Physiol.

[17] Ong J, Tavakkoli A, Zaman N et al (2022) Terrestrial health applications of visual assessment technology and machine learning in spaceflight associated neuro-ocular syndrome. npj Microgravity 8(1):37. 10.1038/s41526-022-00222-7 10.1038/s41526-022-00222-7 PMC9411571 36008494

[18] Waisberg E, Ong J, Zaman N, Kamran SA, Lee AG, Tavakkoli A (2022). Head-mounted dynamic visual acuity for G-transition effects during interplanetary spaceflight: technology development and results from an early validation study. Aerosp med hum perform.

[19] Ong J, Zaman N, Kamran SA (2022). A multi-modal visual assessment system for monitoring spaceflight associated neuro-ocular syndrome (SANS) during long duration spaceflight. J Vis.

[20] Waisberg E, Micieli JA (2021). Neuro-ophthalmological optic nerve cupping: an overview. EB.

